# Precision of SPECT/CT Allows the Diagnosis of a Hidden Brodie’s Abscess of the Talus in a Patient with Sickle Cell Disease

**DOI:** 10.1007/s13139-014-0311-3

**Published:** 2014-12-03

**Authors:** Hassan Al-Jafar, Eman Al-Shemmeri, Jehan Al-Shemmeri, Leena Aytglu, Uzma Afzal, Saud Al-Enizi

**Affiliations:** 1 Hematology Department, Amiri Hospital, Kuwait City, State of Kuwait; 2 Nuclear Medicine Department, Farwaniya Hospital, Al-Farwania City, State of Kuwait; 3 Faculty of Medicine, Nuclear Medicine Department, Kuwait University, Kuwait, Kuwait; 4 Molecular Imaging Center, Jaber Al-Ahmad Center, Kuwait City, Kuwait; 5 Nuclear Medicine Department, Farwaniya Hospital, Al-Farwania City, State of Kuwait

**Keywords:** Sickle cell disease, Brodie’s abscess, SPECT-CT

## Abstract

Brodie’s abscess is a rare subacute osteomyelitis that can be found in sickle cell disease along with other bone complications. A 21-year-old female with sickle cell disease was presenting frequently to the medical casualty department for painful vasoocclusive crises and for persistent ankle pain and swelling. Hybrid imaging with single-photon emission computed tomography-computed tomography (SPECT-CT) incidentally revealed Brodie’s abscess in the talus bone of the ankle, causing persisting long-standing pain. SPECT-CT is a modern technology used to scan bone to detect both anatomical and functional abnormalities with high specificity. Brodie’s abscess is a rare bone inflammation that could be a hidden cause of pain and infection in sickle cell disease. Although rare, this lesion requires more attention in patients with sickle cell disease because their immunocompromised status renders them prone to this infection.

## Introduction

Brodie’s abscess, first described by Sir Benjamin Brodie in 1832 [1], is a rare bone lesion variant of subacute osteomyelitis with a presentation that is atypical and usually late [2, 3]. Diagnosis is difficult because the characteristic signs and symptoms of the acute form of the disease are absent [4]. The lesion is caused by organisms that reach the bone from a disrupted site elsewhere in the body, such as a skin pustule, furuncle, impetigo, or an infected blister or burn, or secondary to an infection of another organ system (urogenital infections, enteritis, cholangitis, or endocarditis) [5]. Brodie’s abscess is a localized walled-off abscess that commonly involves the metaphysis of the long bones of the lower extremities and seldom involves regions such as the pelvis, vertebrae, clavicles, or small bones such as the tarsal bones [6].

Sickle cell disease (SCD) is a congenital hemolytic anemia caused by inheritance of abnormal hemoglobin genes and is characterized by acute episodes of painful vasoocclusive crises (VOC) [7]. Homozygous SCD patients have repeated splenic infarctions leading ultimately to autosplenectomy over many years, and patients with the SCD variant sickle cell thalassemia develop splenomegaly and hypersplenism, which require surgical splenectomy. Both autosplenectomy and surgical splenectomy render SCD patients susceptible to various types of infections, mainly from encapsulated bacteria such as nonenteric *Salmonella typhi* spp., which could cause osteomyelitis due to an abnormality in activation and fixation of C3 secondary to hyposplenic function [8]. Recurrent vascular infarctions in SCD cause end-organ damage to the bone, lung, liver, kidney, and skin, making these sites susceptible to infections by unusual organisms [9].

**Fig. 1:**
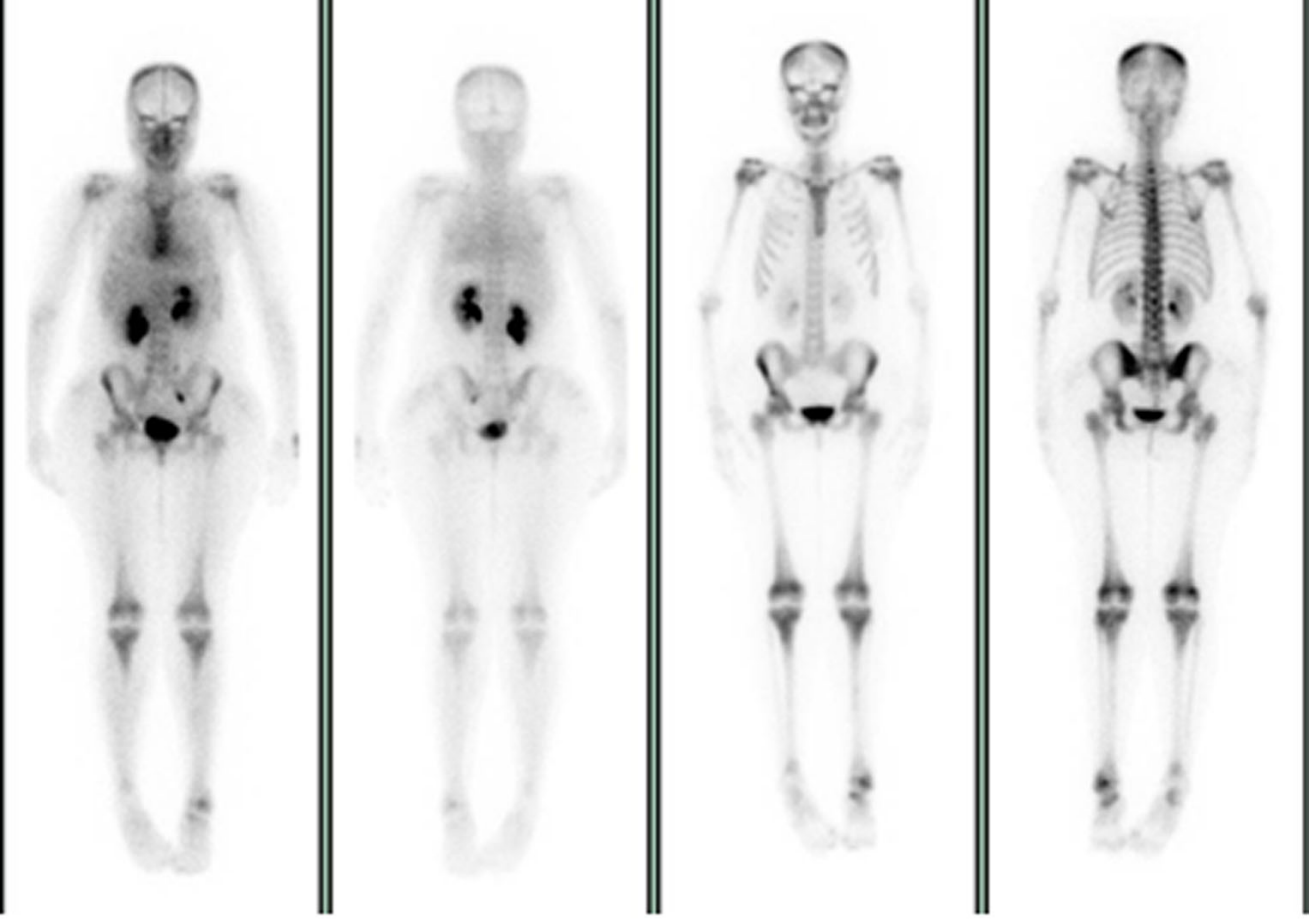
^99m^Tc-methyl diphosphonate whole-body scan showing increased blood pooling and bone uptake involving the proximal talus and region of the left medial malleolus with focal increased uptake in the talus. The increased uptake seen in the shoulders, knees, and sternum is consistent with bone marrow expansion around the large joints

**Fig. 2:**
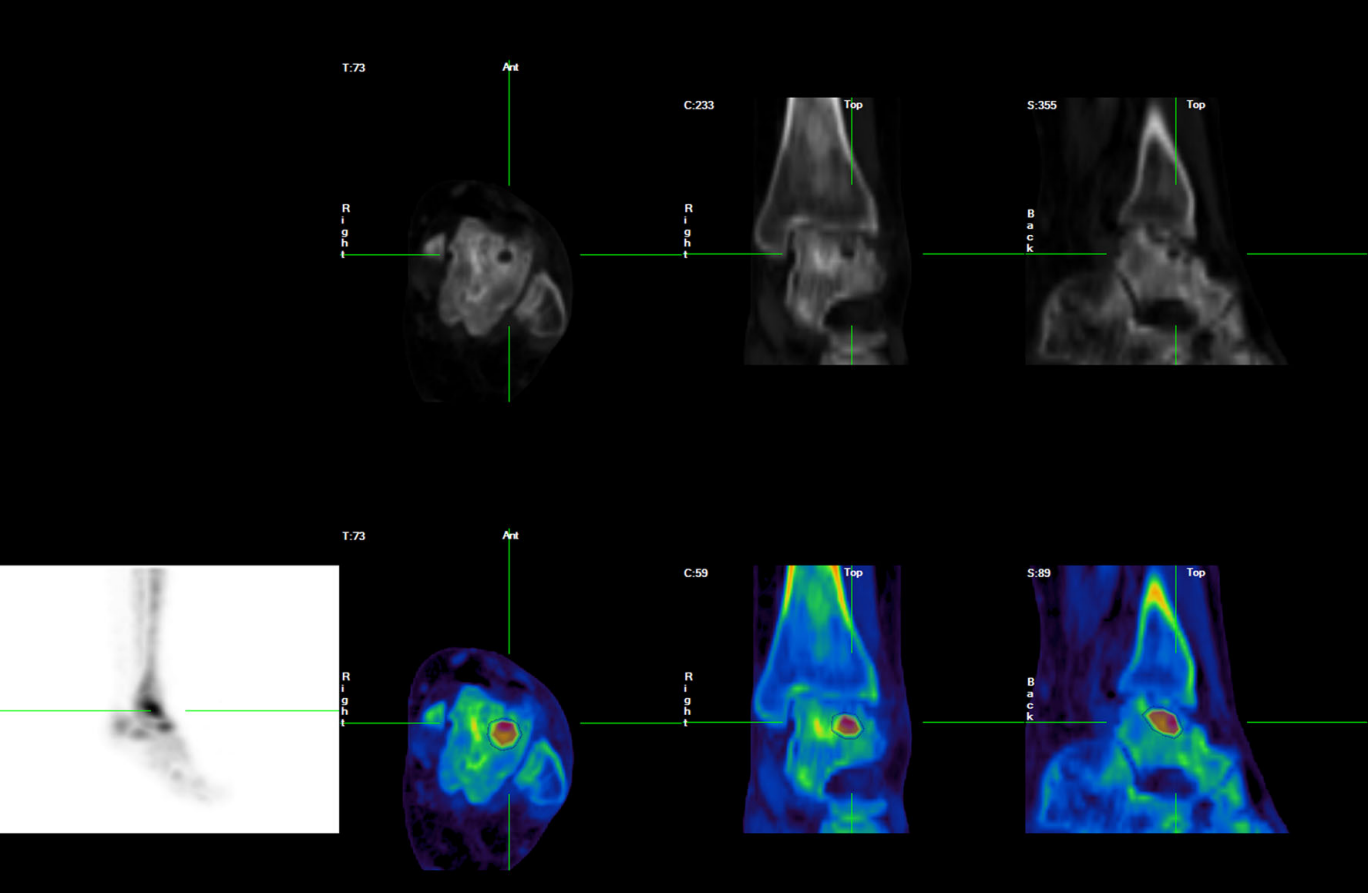
SPECT-CT images show a well-defined lesion with a central radiolucent area surrounded by a dense sclerotic rim and sinus tract formation together with a fracture line in the proximal left talus. These findings are consistent with a diagnosis of Brodie’s abscess in the proximal left talus

SPECT/CT, defined as the fusion of two or more synergistic imaging modes, is a newly developing technology [10], exemplified by single-photon emission computed tomography-computed tomography (SPECT-CT), which combines functional and anatomical data to improve the sensitivity and specificity of detecting and localizing an infectious or tumor process [11]. Hybrid imaging in SPECT-CT and positron emission tomography-CT has become the state of the art in soft-tissue chronic infection diagnosis, and given its ability to allow precise anatomical localization and optimal characterization of abscess formation, it is increasingly used in several fields of nuclear medicine [12, 13]. We describe here the merits of SPECT-CT in the detection of a difficult-to-diagnose rare bone lesion in a patient with SCD (Figs. 1 and 2).

## Case Report

A 21-year-old woman with SCD presented frequently to the medical casualty department with severe pain due to sickle cell VOC. For the last 2 years, her sickle cell pain included intermittent pain in the left ankle. Her recurrent severe VOCs were treated with opioids, as they are the general treatment in such patients. She had experienced osteomyelitis in the right ulnar bone 12 years previously, which resolved after intravenous broad-spectrum antibiotic treatment. Additionally, she underwent splenectomy 4 years ago to reduce blood transfusion requirements because of her congenital hemolytic anemia. She was referred for a triple-phase bone scan and complementary bone marrow and infection imaging as part of the SCD research project. A plain radiograph of the left foot was normal, but SPECT-CT revealed an increased uptake in the shoulders, knees, and sternum on blood pool and delayed images, consistent with marrow expansion. A similar uptake pattern was seen during bone marrow study, confirming bone marrow expansion around the large joints. There was a mildly increased blood pool and bone uptake in the left ankle involving the proximal talus and the region of the medial malleolus, with focal increased uptake in the left talus. This nidus of activity appeared to correspond to a round lucent area surrounded by a dense sclerotic rim and sinus tract formation together with a fracture line in the proximal talus on CT. The WBC scan showed no evidence of significant uptake, and the bone marrow scan showed asymmetrical uptake between the two ankles without a cold lesion in the left talus, which is consistent with moderate bone marrow expansion. The CT and bone scan findings suggested Brodie’s abscess in the proximal left talus with possible surrounding chronic osteomyelitis. Intravenous antibiotics were administered for 10 days with no response. The abscess was then treated with surgical curettage under general anesthesia and antibiotic administration for 10 days. At 18 months after removal of the abscess, the patient did not have pain or swelling at the site of the abscess even with sickle cell VOC.

## Discussion

In addition to the severe frequent painful crises in SCD, Brodie’s abscess could be another hidden cause of severe pain in SCD patients. This rare subacute osteomyelitis usually has an insidious onset with mild signs and symptoms with no systemic reaction or specific laboratory test results. Differential diagnoses include Langerhans cell histiocytosis, osteoid osteoma, intracortical hemangioma, ossifying fibroma, and other infections such as tuberculosis, resulting in delayed diagnosis and management [14]. Brodie’s abscess, accompanied by minimal or absent periosteal reaction, may be so small that detection on a plain radiograph is not possible [15].

The recurrent pain crises in SCD and the changing sites of pain in these crises make clinical diagnosis of such cases extremely difficult. Infarction episodes in vasoocclusive crises can be associated with swelling, hotness, and tenderness, which can lead to missing the diagnosis of subacute osteomyelitis. The new imaging technology of hybrid scintigraphy by SPECT-CT has become an important investigative tool to assess the different complicated bone lesions in SCD, commonly osteopenia, osteoporosis, avascular necrosis, and pathological fracture. Hybrid imaging, a newly developing technology, improves the diagnostic performance of scintigraphic techniques by avoiding false-positive or equivocal results and provides both anatomical and functional details [16]. It also improves the quality of interpretation and increases specificity by obtaining more accurate imaging data [17].

## Conclusion

We describe a case in which an important finding was discovered incidentally, using hybrid imaging (SPECT-CT), at the start of a massive research project to assess bone involvement in SCD patients in our country. We believe this finding should serve to increase our awareness of another new painful and destructive bone lesion, which may not be detected by plain radiography, in addition to a number of other catastrophic bone complications in SCD.
